# The Gut–Endometrium Axis: Exploring the Role of Microbiome in the Pathogenesis and Treatment of Endometrial Cancer—A Narrative Review

**DOI:** 10.3390/cancers17061044

**Published:** 2025-03-20

**Authors:** Beibei Zhang, Nur Fatin Nabilah Mohd Sahardi, Wen Di, Xiaoran Long, Mohamad Nasir Shafiee

**Affiliations:** 1 Department of Obstetrics and Gynaecology, Faculty of Medicine, Universiti Kebangsaan Malaysia, Kuala Lumpur 56000, Malaysia; p125922@siswa.ukm.edu.my; 2 Faculty of Medicine, Universiti Kebangsaan Malaysia, Kuala Lumpur 56000, Malaysia; nurfatinnabilah@ukm.edu.my; 3 Department of Obstetrics and Gynecology, Renji Hospital, School of Medicine, Shanghai Jiao Tong University, Shanghai 200127, China; diwen163@163.com (W.D.); x.long@sjtu.edu.cn (X.L.)

**Keywords:** endometrial cancer, gut microbiome, endometrial microbiome

## Abstract

**Background/Objectives**: Endometrial cancer (EC) is a prevalent gynecological malignancy with an increasing incidence, particularly in developed countries. Recent research has demonstrated the significant involvement of gut and endometrial microbiomes in the pathogenesis and progression of EC. This review provides a comprehensive overview of the existing knowledge on the interactions between these microbial communities and their influence on EC. **Methodology**: A literature review was conducted using electronic databases including Google Scholar, Scopus, and PUBMED, covering the period from 2017 to 2024. The following keywords were used for the literature search: (1) gut microbiome and endometrial cancer, (2) endometrium microbiome and endometrial cancer, and (3) endometrial cancer and microbial dysbiosis. The selected articles were chosen based on inclusion and exclusion criteria. Scale for Assessment of Narrative Review Articles (SANRA) was used for evaluating and assessing the quality of articles. **Results**: The gut microbiome modulates systemic inflammation, immune responses, and estrogen metabolism, all of which are crucial factors in EC development. Dysbiosis is an imbalance in the composition of microbes that can cause chronic inflammation and hormonal imbalances, which can contribute to the EC. Similarly, the endometrial microbiome, while less extensively studied, has been implicated in EC through mechanisms involving local immune modulation and the production of harmful metabolites. Probiotics, prebiotics, fecal microbiota transplantation (FMT), and personalized microbiota-based therapies can be used as clinical interventions for EC management. This review emphasizes the need for further research to explore the gut–endometrium axis and its potential for innovative therapeutic approaches. Understanding these complex interactions will become a novel strategy to prevent and treat EC, ultimately enhancing patient outcomes.

## Simple Summary

Endometrial cancer (EC) is a common gynecological malignancy, especially in developed countries. Emerging research emphasizes the critical roles of gut and endometrial microbiomes in EC pathogenesis and progression. The gut microbiome impacts systemic inflammation, immune responses, and estrogen metabolism. Dysbiosis or microbial imbalance can lead to chronic inflammation and hormonal disruptions, fostering a pro-tumorigenic environment in the endometrium. The endometrium microbiome may contribute to EC through local immune modulation and harmful metabolite production. This review underscores the importance of further research into the gut–endometrium axis to develop novel preventive and therapeutic strategies aiming to enhance patients’ outcomes in EC care.

## 1. Introduction

Endometrial cancer (EC) ranks as the fourth most common cancer in women, exhibiting notable disparities in incidence and mortality across different racial and ethnic groups [1]. According to recent statistics, between 2014 and 2018, the overall incidence rate of EC stood at 27.7 per 100,000, with a five-year survival rate of 81.2%. However, this figure drops significantly to 63.4% among non-Hispanic Black women, highlighting a concerning disparity in outcomes based on racial and ethnic background [2].

EC is particularly prevalent in developed countries, and its incidence is on the rise, closely linked to increasing rates of obesity and aging populations. Primary risk factors for EC include unopposed estrogen exposure, obesity, diabetes, hypertension, and genetic predispositions [3]. Obesity, in particular, has a profound impact on EC risk due to the increased production of estrogen from adipose tissue. Additionally, conditions such as polycystic ovary syndrome (PCOS) and metabolic syndrome further exacerbate the risk of developing EC [4,5,6]. While early-stage EC typically has a favorable prognosis when diagnosed and treated promptly, advanced or recurrent EC poses significant treatment challenges and is associated with poor outcomes [1]. Treatment options for advanced EC are limited and often involve a combination of surgery, radiation therapy, and chemotherapy, which can be accompanied by severe side effects and limited efficacy [7].

Recent research has begun to elucidate the complex relationship between the human microbiome and various cancers [8]. The human microbiome, which encompasses diverse communities of microorganisms residing in the body, is essential for maintaining homeostasis and modulating the immune system [9]. Dysbiosis, or disruption of microbiome balance, has been implicated in the pathogenesis of several cancers [10]. The gut microbiome significantly influences cancer formation and progression by modulating inflammatory responses, affecting metabolic processes, and altering the immunological environment [11]. Studies have demonstrated that an imbalance in the gut microbiome might induce chronic inflammation, a known risk factor for cancer, by enhancing the synthesis of pro-inflammatory cytokines and diminishing the production of anti-inflammatory compounds [10].

The endometrial microbiome is essential for maintaining reproductive health and immune homeostasis [12]. However, dysbiosis within the endometrial microbiome has been associated with persistent inflammation, immune disruptions, and an increased risk of cancer [13]. Despite these findings, the exact role of the gut and endometrial microbiota in the progression of EC remains poorly understood. Additionally, there is limited knowledge on the potential of microbiome-based interventions for early detection, prevention, and treatment, highlighting a critical gap in current research.

This review aims to synthesize evidence on the mechanisms by which the gut and endometrial microbiota contribute to the pathogenesis of EC, as well as explore potential microbiome-based interventions. By synthesizing recent findings, we seek to highlight the emerging role of the microbiome in EC etiology, suggest novel diagnostic and treatment approaches, and identify key research gaps. Understanding these complex microbial interactions could establish the way for innovative strategies in EC management, ultimately improving patient outcomes.

## 2. Methods

### 2.1. Search Strategy

An extensive literature search was performed to identify relevant articles exploring the role of the microbiome in the pathogenesis and treatment of EC via the gut–endometrium axis. Published and full-text articles were obtained from various electronic databases such as Google Scholar, Scopus, and PUBMED from the year 2017 until 2024. The literature search was conducted using the related keywords: (1) gut microbiome and endometrial cancer, (2) endometrium microbiome and endometrial cancer, and (3) endometrial cancer and microbial dysbiosis. All English language articles relevant to the review’s objective, as identified through these keywords, were included in this review.

### 2.2. Search Criteria

The inclusion criteria were (i) clinical/preclinical studies, (ii) study on gut microbiota and its effects on endometrial cancer, and (iii) free-text articles available in English only. The exclusion criteria were (i) gray literature (unpublished academic paper), (ii) conference and abstract publication, (iii) study not involving the gut microbiota or endometrial cancer, and (v) non-English article.

### 2.3. Quality Assessment

The reporting of this study adheres to the guideline of the Scale for the Assessment of Narrative Review Articles (SANRA) tool, a concise guideline for critically evaluating nonsystematic articles [14]. This tool covers six items: (1) explanation of the review’s importance, (2) statement of the aims, (3) description of the literature search, (4) referencing, (5) scientific reasoning, and (6) appropriate presentation of endpoint data.

## 3. Pathophysiology of Endometrial Cancer

EC is primarily divided into two distinct types: Type I and Type II. Type I, or endometrioid carcinoma, is estrogen-dependent and generally has a better prognosis, accounting for about 80% of cases. It often arises in the setting of endometrial hyperplasia, a condition characterized by the thickening of the endometrium due to prolonged estrogen exposure without the counterbalancing effect of progesterone [15]. This prolonged estrogen exposure can result from various factors, including obesity, PCOS, and hormone replacement therapy. Type II, which includes serous, clear cell, and carcinosarcoma subtypes, is less common, tends to be more aggressive, and is not associated with estrogen exposure [16]. These carcinomas often occur in a background of atrophic endometrium and are characterized by distinct molecular alterations compared to Type I EC, making them more resistant to conventional therapies.

The progression of EC typically involves a sequence from normal endometrium to hyperplasia and finally to carcinoma, with increasing genetic and molecular abnormalities at each stage [17]. In Type I EC, this progression is often driven by mutations in genes. One of them is *PTEN*, a tumor suppressor gene that is commonly mutated in endometrial hyperplasia and early-stage carcinomas [18]. Other common genetic alterations in Type I EC include mutations in the *PIK3CA* gene, which encodes a subunit of the phosphoinositide 3-kinase (PI3K) enzyme, and in the *ARID1A* gene, involved in chromatin remodeling. These mutations contribute to the uncontrolled proliferation of endometrial cells and the development of cancer [19]. Type II EC, on the other hand, is associated with different molecular changes, including mutations in the *TP53* gene, which encodes the p53 tumor suppressor protein, and the *HER2/neu* oncogene. These alterations are linked to more aggressive tumor behavior and poorer clinical outcomes [19]. Understanding these molecular differences is crucial for developing targeted therapies that can effectively treat each subtype of EC.

The major risk factors for EC encompass hormonal, genetic, and environmental influences. Unopposed estrogen exposure, obesity, diabetes, hypertension, and a family history of cancer are significant contributors [20]. Obesity, in particular, is a well-established risk factor due to the increased production of estrogen from adipose tissue. Adipose tissue converts androstenedione to estrone, a form of estrogen, through the action of the enzyme aromatase. This estrogenic effect is particularly pronounced in postmenopausal women, where the ovaries no longer produce significant amounts of estrogen, and adipose tissue becomes the main source of the hormone [21].

Diabetes and hypertension have been linked with an increased risk of EC, likely due to the associated metabolic disturbances and chronic inflammation that can promote cancer development. Insulin resistance, prevalent in obese individuals and those with type 2 diabetes, may result in elevated levels of insulin and insulin-like growth factors, which have mitogenic and anti-apoptotic effects on endometrial cells, further increasing cancer risk [22].

Genetic predisposition plays a crucial role in EC, with hereditary conditions like Lynch syndrome (hereditary nonpolyposis colorectal cancer (HNPCC)) significantly increasing the risk. Individuals with Lynch syndrome have mutations in DNA mismatch repair (MMR) genes, including *PMS2*, *MLH1*, *MSH2*, and *MSH6*, which are critical for DNA repair. Loss of function in these genes leads to instability of microsatellite and an increased risk of various cancers, including EC. Genetic mutations in other genes, such as *PTEN*, *POLE*, and *WNT4*, are important contributors to EC pathogenesis and progression [23]. Additionally, common genomic polymorphisms add to the overall risk, making genetic screening an essential tool in risk assessment and management [24].

Environmental factors, including lifestyle and dietary habits, significantly influence the risk of EC. Obesity, associated with increased estrogen production, is a well-established risk factor. Diets high in fat and low in fiber can contribute to obesity and, consequently, to higher estrogen levels. Furthermore, exposure to endocrine disruptors such as bisphenol A (BPA) and diethylstilbestrol (DES) has been associated with an increased risk of EC [25]. These chemicals can mimic estrogen in the body, binding to estrogen receptors and promoting cell proliferation in the endometrium.

The role of chronic inflammation in the development of EC is also gaining attention. Conditions such as chronic endometritis, pelvic inflammatory disease, and even long-term use of intrauterine devices can lead to persistent inflammation in the endometrium. Chronic inflammation can result in the production of reactive oxygen species (ROS) and pro-inflammatory cytokines, which can contribute to DNA damage and promote carcinogenesis [26].

Understanding the complex interplay of these risk factors is crucial for establishing effective preventative and treatment approaches. This complexity extends to the role of the gut and endometrial microbiome, which are increasingly acknowledged as significant factors in the pathogenesis of EC. Transitioning to a discussion on microbiomes illuminates how these microorganisms influence the development and progression of EC, potentially offering new avenues for intervention and management.

## 4. Gut Microbiome and Endometrial Cancer

The human gut microbiome, a complex ecosystem comprising trillions of microorganisms, is crucial for general health by aiding digestion, synthesizing essential vitamins, and regulating the immune system. Five bacterial phyla are predominantly in the gut microbiome, which are Firmicutes, Bacteroidetes, Proteobacteria, Actinobacteria, and Verrucomicrobia [27]. Among these, Bacteroidetes and Firmicutes are the most dominant phyla in the human gut, followed by Actinobacteria (e.g., *Bifidobacterium*) and Proteobacteria (e.g., *Enterobacteria*). Verrucomicrobia is the least abundant phylum in gut microbiota, as represented by *Akkermansia* [28].

Each person has a unique and diverse microbiota shaped by various factors such as diet, genetics, medication use, and lifestyle [29]. Among these, diet is the most significant factor in determining and influencing an individual’s microbiota composition. Diet and microbiota can work together to influence host physiology through immune interaction and the production of certain metabolites [30]. The Mediterranean diet, for example, can lead to the production of specific metabolites such as SCFAs [31]. These SCFAs are essential for providing energy, acting as signaling molecules, improving insulin sensitivity, reducing fat accumulation, supporting the immune system, and enhancing gut motility and nutrient absorption [32]. The microbiota can also be influenced by prebiotic fibers and bacteriocins, especially those produced by lactic acid bacteria [33]. These compounds affect the host’s physiology and health through two mechanisms. First, exploitative competition promotes the growth of beneficial bacteria by allowing them to utilize available nutrients efficiently. Then, interference competition inhibits the growth of harmful bacteria and pathogens through direct antagonistic interactions.

Additionally, obesogenic diets, including high-fat, high-calorie, high-fructose intake and frequent meals, can alter gut microbiota composition and compromise intestinal wall integrity [34]. In contrast, a diet rich in fiber and probiotics supports a healthy gut microbiome, which may have protective effects against endometrial carcinogenesis by reducing systemic inflammation and maintaining balanced estrogen levels [35]. Gut bacteria ferment dietary fibers to produce short-chain fatty acids (SCFAs), which possess anti-inflammatory properties and play a crucial role in immune modulation. Conversely, a high-fat diet is associated with an increased prevalence in Gram-negative bacteria, leading to elevated lipopolysaccharide (LPS) levels. Studies have shown that a high fat diet induces gut dysbiosis, contributing to systemic inflammation and altered estrogen metabolism, thereby increasing the risk of EC [36].

Li et al. [37] reported a significant reduction in alpha diversity of gut microbiota in EC patients as compared to healthy control. They found that *Clostridia*, *Firmicutes*, *Clostridiales*, *Faecalibacterium*, *Ruminococcaceae*, and *Gemmiger_formicis* were markedly reduced in EC patients, while *Gammaproteobacteria*, *Enterobacteriales*, *Proteobacteria*, *Shigella*, and *Enterobacteriaceriaceae* were significantly elevated. Among these, *Proteobacteria*, *Enterobacteriaceae*, *Enterobacteriales*, and *Shigella* were identified as the dominant gut microbiota in EC patients.

Additionally, certain gut microbiota species are known producers of β-glucuronidase enzyme. This enzyme deconjugates estrogen metabolites in the gut, allowing their reabsorption into circulation [38]. Gut dysbiosis can lead to an overabundance of β-glucuronidase- producing bacteria, potentially resulting in excessive estrogen level production. Notably, the phylum Bacteroidetes exhibits a greater diversity and abundance of β-glucuronidase-producing bacteria [38].

In conclusion, the gut microbiome plays a significant role in the initiation and progression of EC through multiple mechanisms, including immune modulation, production of metabolites, and modification of the inflammatory environment. Understanding the complex interactions between the gut microbiome and cancer may establish the way for novel therapeutic strategies aimed at restoring microbial balance and preventing cancer progression.

## 5. Endometrial Microbiome and Endometrial Cancer

The endometrial microbiome, though less extensively studied than the gut microbiome, has appeared as a critical factor in the pathogenesis of EC. This unique community of microorganisms within the uterus serves a crucial function in preserving reproductive health and immune homeostasis. Recent research has begun to unravel the complex relationship between the endometrial microbiome and EC, suggesting that dysbiosis in this microbial community may lead to the development and progression of the disease.

Previously, the presence of the endometrial microbiome was once considered improbable due to the belief that the uterus was a sterile environment. However, advancements in sequencing technologies have revealed that the endometrium hosts a diverse array of bacteria, including species such as *Lactobacillus*, *Gardnerella*, and *Atopobium* [39,40]. Moreno et al. [41] highlighted that the endometrium is primarily dominated by *Lactobacillus* sp., followed by *Gardnerella*, *Bifidobacterium*, *Prevotella*, and *Bifidobacterium*. Another study found that *Lactobacillus*, *Gardnerella*, *Bifidobacterium*, *Streptococcus*, and *Alteromonas* were more common in healthy endometrial tissue as compared to patients with endometrial polyps and chronic endometritis [42].

However, dysbiosis in the endometrial microbiome, characterized by an imbalance between beneficial and pathogenic bacteria, has been implicated in various reproductive disorders, including EC. A study by Morenoet al. [41] displayed that depletion of *Lactobacillus* species and the presence of certain pathogenic bacteria such as *Bifidobacterium*, *Klebsiella*, and *Gardnerella* in endometrial fluid and endometrial biopsy samples were associated with unsuccessful reproductive outcomes. This was supported by another study which found that a reduction in *Lactobacillus*, particularly *L. cripatus*, led to increased bacterial diversity and an abundance of *Prevotella*, *Peptoniphilus*, *Porphyromonas*, and *Anaerococcus* in EC patients [43]. Lu et al. [44] further highlighted that local microflora diversity in EC patients was reduced, with a notable increase in *Micrococcus* species compared to patients with benign uterine lesions. This suggests a potential role of *Micrococcus* in EC development [44]. A systematic review by Stabile et al. [45] identified *Actinobacteria*, *Bacteroidetes*, *Firmicutes*, and *Proteobacteria* as the dominant bacterial phyla in EC patients, comprising both Gram-positive and Gram-negative anaerobic bacteria.

The composition of these endometrial microbiota is influenced by several factors, including hormonal regulation, reproductive and gynecological conditions, and antibiotic use, together with lifestyle and environmental factors. Estrogen and progesterone fluctuation during the menstrual cycle significantly impact microbial diversity in the endometrium [46]. Estrogen promotes the *Lactobacillus* colonization, whereas elevated progesterone levels lead to greater microbial variability [47]. Consequently, postmenopausal women often display a lower abundance of *Lactobacillus*, which may contribute to the increased of inflammation and susceptibility to infections. A study conducted by Walsh et al. [47] displayed that *Anaerococcus lactolyticus*, *Anaerococcus tetradius*, *Campylobacter ureolyticus*, and *Peptoniphilus coxii* species associated with the status of menopause in EC patients. Additionally, antibiotics and probiotics can disrupt the microbial balance by reducing both beneficial and harmful bacteria, potentially promoting the overgrowth of pathogenic species [48].

In conclusion, the endometrial microbiome, though less extensively studied than the gut microbiome, plays a significant role in maintaining reproductive health and immune homeostasis. Emerging evidence highlights that dysbiosis within this endometrial microbiome may contribute to the pathogenesis of EC.

## 6. Interaction Between Gut Microbiome and Endometrial Microbiome in Endometrial Cancer

**Figure 1:**
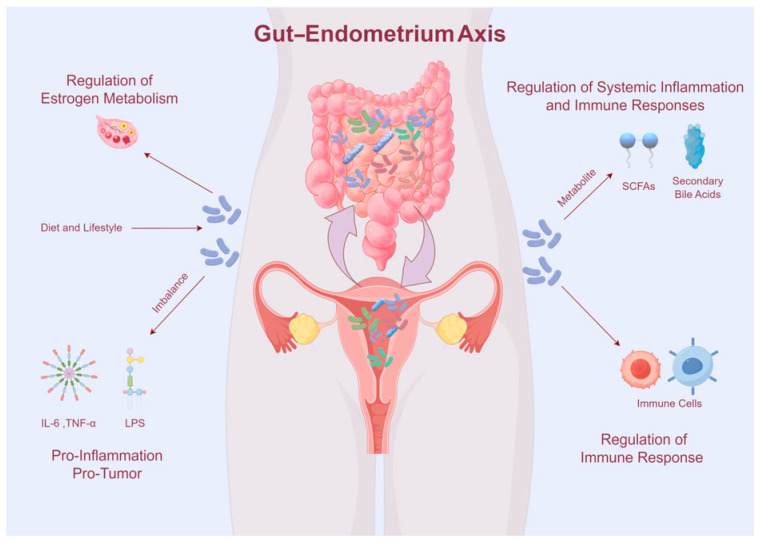
Interaction between the gut microbiome and endometrial microbiome.

The interaction between the gut microbiome and endometrial microbiome, often denoted as the “gut–endometrium axis”, plays a crucial role in the development and progression of EC. This complex interplay involves systemic interactions mediated by microbial metabolites, immune modulators, and hormonal regulation, influencing both local and distant microenvironments (Figure 1).

The gut microbiome plays a crucial role in cancer development by modulating the host’s immune system, which enhances the development and function of immune cells, as well as impacts the body’s capability to combat cancer. These interactions are particularly relevant in the context of EC, where dysbiosis, or the imbalance of the gut microbiome, has been increasingly linked to disease formation [8]. Research by Chen et al. [49] demonstrated that gut microbiome dysbiosis can alter systemic immune responses, which in turn affect the endometrial microenvironment, contributing to the development of EC. Previous studies have shown that the Firmicutes/Bacteroidetes ratio and the abundance of the Bifidobacterium phylum increased in endometriosis [36]. However, the gut microbiome composition in EC is differs significantly from that healthy control [37]. EC patients exhibit a marked reduction in *Firmicutes*, *Clostridia*, *Clostridiales*, *Ruminococcaceae*, *Faecalibacterium*, and *Gemmiger formicis*, while *Proteobacteria*, *Gammaproteobacteria*, *Enterobacteriaceae*, and *Shigella* are significantly elevated.

Dysbiosis of gut microbiome can lead to chronic inflammation, a well-established risk factor for various cancers, including EC [8]. Dysbiosis compromises intestinal barrier integrity, leading to increased intestinal permeability and allows microbial components such as lipopolysaccharides (LPS) to enter the systemic circulation [50]. LPS activates Toll-like receptor 4 (TLR4) signaling which triggers a pro-inflammatory cytokine cascade, leading to elevated of IL-6, TNF-α, and IL-1β [51]. These cytokines activate various signaling pathways, promoting cell proliferation, inhibiting apoptosis, and enhancing angiogenesis, all of which are key processes in cancer development. Chronic inflammation induced by an imbalanced microbiome can create a microenvironment conducive to tumorigenesis by promoting cellular proliferation, inhibiting apoptosis, and facilitating angiogenesis [10]. Studies have demonstrated that microbial profiles associated with chronic inflammatory conditions correlate with an increased risk of EC [38]. For instance, microbial communities that enhance the generation of pro-inflammatory cytokines can exacerbate the inflammatory milieu, contributing to tumorigenesis. Additionally, persistent low-grade inflammation is a hallmark of cancer, and the gut microbiome may affect systemic inflammation by producing pro-inflammatory cytokines and other inflammatory mediators [41]. Specific gut bacteria, such as *Fusobacterium nucleatum*, have been associated with increased inflammation and are linked to colorectal cancer, suggesting a similar mechanism could be at play in EC [42]. The chronic inflammation mediated by gut dysbiosis can disrupt normal cellular functions and promote malignant transformations in endometrial tissue.

Recent research has emphasized the gut microbiome’s influence on systemic inflammation and immune responses, which can indirectly affect the endometrial environment. The gut microbiome produces a variety of metabolites, such as SCFAs, microbial-associated molecular patterns (MAMPs), and secondary bile acids that may enter the bloodstream and have systemic effects. Feng et al. [52] highlighted the role of microbial metabolites in modulating systemic inflammation and its potential impact on endometrial carcinogenesis. SCFAs, particularly butyrate, possess anti-inflammatory characteristics and regulate the immune response, possibly influencing the endometrial microenvironment [53]. Butyrate, in particular, has been shown to enhance the regulatory T cells (Tregs), which help preserve immune tolerance and reduce inflammation, thus potentially providing a protective effect against EC [54,55]. Conversely, secondary bile acids produced by gut bacteria have been implicated in promoting carcinogenesis by inducing DNA damage and supporting a pro-inflammatory environment, thereby affecting EC risk [56]. These bile acids can activate signaling pathways, including the nuclear factor–kappa B (NF-κB) pathway, which is known to be involved in inflammation and cancer development [57].

Moreover, the gut microbiome is integral to estrogen metabolism, which is vital for endometrial maintenance and function. Certain gut microbiota, such as *Streptococcus agalactiae*, *Bacteroides fragilis*, and *Escherichia coli*, synthesize beta-glucuronidase, an enzyme that deconjugates estrogen metabolites, making them available for reabsorption into the bloodstream [58]. This process can increase systemic estrogen levels, thereby potentially promoting estrogen-dependent cancers such as EC [7,36]. Estrogen is essential in modulating the microenvironment of the lower female reproductive system by increasing glycogen concentration, increasing epithelial thickness, increasing mucus secretion, and promoting the abundance of lactobacilli, which indirectly contribute to lactic acid production. Studies have shown that women with EC have distinct gut microbiome profiles compared to healthy controls, with an overrepresentation of bacteria capable of producing beta-glucuronidase [43]. This alteration in estrogen metabolism due to gut microbiota dysbiosis underscores the systemic impact of gut health on EC risk. Elevated levels of estrogen have been linked to increased cellular proliferation and reduced apoptosis, contributing to the pathogenesis of EC.

Systemic inflammation and immune suppression mechanisms further facilitate dysbiosis of the endometrial microbiome. Under normal conditions, *Lactobacillus* species exert protective effects by promoting a balanced endometrial microbiome and reducing inflammation. However, dysbiosis of the endometrial microbiome promotes the reduction in *Lactobacillus* and an overgrowth of *Atopobium*, *Fusobacterium*, and *Garderella*. Functionally active microorganisms in the endometrium can interact with host gene regulation, influencing tumor occurrence and migration, underscoring the microbiome’s importance in EC treatment and prognosis [53]. Studies have revealed significant differences in cervicovaginal microbiome composition between EC patients and those with benign conditions, with specific microorganisms like *Mobiluncus curtisi* and *Dialister pneumosintes* implicated in promoting endometrial carcinogenesis [54].

Beyond inflammation, the endometrial microbiome influences cancer development through the production of microbial metabolites. These metabolites effect cellular processes, including cell proliferation, apoptosis, and DNA repair mechanisms. Some bacterial species produce metabolites that directly damage DNA or promote oxidative stress, contributing to carcinogenesis [48,50]. Dysbiosis of the endometrial microbiota is associated with increased inflammatory cytokines, including interleukin-6 (IL-6) and interleukin-17 (IL-17), which promote the development and progression of EC. Specific bacteria like *Micrococcus* have been positively correlated with these markers in EC patients [51].

In conclusion, the interaction between the gut microbiome and the endometrial microbiome is a critical factor in the development and progression of EC. Dysbiosis in the gut microbiome can lead to systemic inflammation, altered estrogen metabolism, and the production of harmful metabolites, all of which can influence the endometrial microenvironment. Understanding these intricate interactions provides new insights into potential therapeutic strategies aimed at modulating the gut–endometrium axis to prevent and treat EC.

## 7. Current Clinical Therapies Targeting the Endometrial and Gut Microbiome in Endometrial Cancer

Recent advancements in microbiome research have opened new avenues for the clinical management of EC. The manipulation of endometrial and gut microbiomes presents a promising therapeutic strategy aimed at modulating the local and systemic environments that contribute to cancer progression. Here, we explore various clinical approaches targeting the microbiome for the treatment and management of EC.

Probiotics and prebiotics have been extensively investigated for their ability to influence the gut microbiome and improve health outcomes. Probiotics, such as *Bifidobacterium* and *Lactobacillus species*, have shown potential in restoring microbial balance and reducing inflammation. Clinical trials have demonstrated that probiotics can enhance the immune response and may reduce the risk of cancer by modulating the gut microbiome composition [53,59]. Prebiotics, which are nondigestible dietary components that enhance the growth of beneficial bacteria, have also been shown to enhance gut health and may have protective effects against cancer development [54]. Studies have shown that the administration of probiotics results in a reduction in pro-inflammatory cytokines and an elevation of anti-inflammatory cytokines, thus modulating the immune response in a way that may reduce cancer risk. Moreover, prebiotics like inulin and fructo-oligosaccharides have been shown to promote the growth of beneficial gut microbiota, resulting in enhanced gut health and potentially lowering the risk of EC through systemic anti-inflammatory effects [55].

FMT is an emerging therapeutic approach that comprises the transplantation of fecal matter from a healthy donor into a patient’s gastrointestinal tract to restore a balanced microbiome. FMT has shown promising results in treating *Clostridium difficile* infections and is being explored for its potential in cancer therapy. By restoring a healthy gut microbiome, FMT may reduce systemic inflammation and modulate immune responses, thereby influencing cancer progression [56]. The success of FMT in other contexts suggests that it could be an effective strategy for altering the gut microbiome to enhance outcomes in patients with EC.

Immunotherapy has revolutionized cancer treatment, and recent studies have emphasized the impact of the gut microbiome on the efficacy of immune checkpoint inhibitors. Specific bacterial species in the gut microbiome are linked with improved responses to immunotherapy. For example, patients with a higher abundance of *Akkermansia muciniphila* in their gut microbiota showed better responses to PD-1 blockade therapy [57,60].

Antibiotic therapy can drastically modify the composition of the gut microbiome, which may have therapeutic implications for cancer. While antibiotics are typically used to treat bacterial infections, they can also be used selectively to deplete harmful bacterial species associated with cancer progression. However, the indiscriminate usage of antibiotics can disrupt the balance of the microbiome and potentially worsen outcomes, highlighting the need for targeted approaches [61,62].

Personalized medicine approaches are gaining traction in the field of oncology, including microbiome-based therapies. These approaches involve tailoring treatments according to an individual’s microbiome profile. Advanced sequencing technology and bioinformatics tools allow for the precise characterization of the microbiome, enabling the development of personalized probiotics and dietary interventions designed to modulate the microbiome in a way that supports cancer treatment and prevention [63].

Future clinical research should focus on large-scale, longitudinal studies to establish causal relationships between microbiome composition and EC progression. Such studies should include diverse populations to assess the impact of genetic and environmental variables on the microbiome and its function in EC. Integrating microbiome research with other omics data, including proteomics, genomics, and transcriptomics, will provide a comprehensive understanding of the complex interactions driving EC.

In conclusion, targeting the endometrial and gut microbiomes represents a promising frontier in the clinical management of EC. Probiotics, prebiotics, FMT, immunotherapy, and personalized microbiome-based therapies are potential strategies to modulate the microbiome and enhance patient outcomes (Table 1). Future research should continue to explore these approaches and their integration into personalized medicine.

## 8. Key Findings and Interpretation

**Figure 2:**
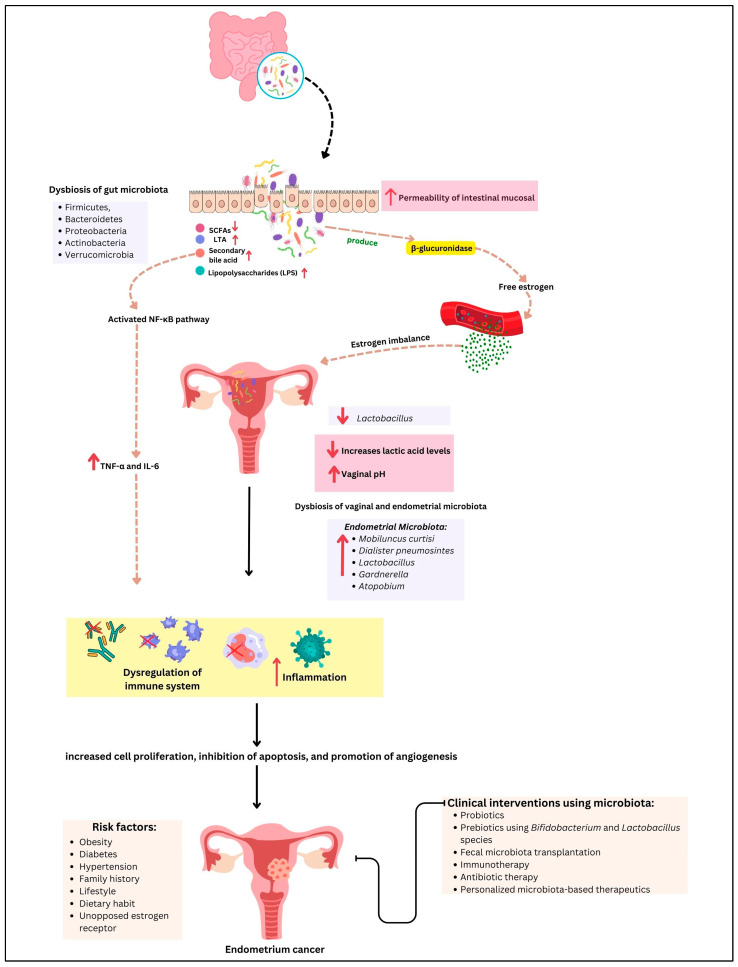
The gut–endometrium axis and its role in EC pathogenesis and treatment. Dysbiosis of the gut microbiota contributes to estrogen imbalance and promotes systematic inflammation via microbial metabolites such as short-chain fatty acids (SCFAs) and lipopolysaccharides (LPS), disrupting immune homeostastis. Simultaneously, a decreased level of Lactobacillus in the vaginal microbiota leads to lower lactic acid production and an increase in vaginal pH, creating an environment conducive to dysbiosis of the endometrial microbiome and immune dysregulation. These factors collectively contribute to tumor initiation and progression. Potential therapeutic interventions, including prebiotics, probiotics, and microbiome-targeted therapies, are emerging strategies for modulating the gut–endometrial axis in the EC management. The arrows ↑ and ↓ indicate an increase or decrease respectively, in biological parameters, microbiota or clinical markers.

The intricate relationship between the gut and endometrial microbiome and the development and progression of EC underscores the importance of microbial balance in maintaining gynecological health. Our review highlights the multifaceted roles of the microbiome in modulating immune responses, influencing metabolic pathways, and shaping inflammatory environments, all of which contribute to EC pathogenesis (Figure 2).

The evidence presented underscores the gut microbiome’s impact on systemic inflammation and immune modulation. Dysbiosis, characterized by an imbalance in microbial communities, can lead to chronic inflammation, a recognized risk factor for various cancers, including EC. The generation of pro-inflammatory cytokines such as TNF-α and IL-6, driven by gut dysbiosis, illustrates a potential mechanism by which systemic inflammation may promote a pro-tumorigenic environment in the endometrium. These cytokines can disrupt normal cellular processes, promote proliferation, and inhibit apoptosis, creating a favorable environment for cancer development.

Equally significant is the role of the gut microbiome in estrogen metabolism. The enterohepatic circulation of estrogens, mediated by gut bacteria, affects estrogen levels and activity, with dysbiosis potentially increasing estrogen bioavailability. This alteration is particularly pertinent to estrogen-dependent cancers like Type I EC, where hormonal imbalances play a crucial role in tumor development. Elevated estrogen levels can lead to increased endometrial proliferation and the potential for malignant transformation, emphasizing the need for a balanced gut microbiome to maintain hormonal homeostasis.

The endometrial microbiome, though less studied, presents another layer of complexity in EC pathogenesis. Dysbiosis within the endometrium, characterized by a reduction in protective *Lactobacillus* species and a rise in pathogenic bacteria, has been related to chronic inflammation and increased cancer risk. The interactions between the endometrial microbiome and local immune responses, coupled with the production of harmful metabolites, further emphasize the need for a balanced microbial environment to prevent malignancy. For example, the presence of harmful bacteria may facilitate the production of ROS and other genotoxic agents, which may damage DNA and trigger carcinogenesis.

Clinical interventions targeting the microbiome offer promising avenues for EC management. Probiotics and prebiotics have shown potential in restoring microbial balance and reducing inflammation, with clinical trials demonstrating enhanced immune responses and reduced cancer risk. Probiotics such as *Bifidobacterium* and *Lactobacillus* can help restore the gut microbiome balance, decrease the generation of pro-inflammatory cytokines, and improve the anti-inflammatory immune response. Prebiotics, on the other hand, encourage the growth of beneficial bacteria by providing substrates for their metabolism, thereby supporting a healthy microbiome.

FMT emerges as a novel approach, capable of restoring a healthy gut microbiome and modulating systemic inflammation. FMT has shown efficacy in addressing recurrent *Clostridium difficile* infections and is now being investigated for its possible use in cancer therapy. By re-establishing healthy gut microbiota, FMT can reduce systemic inflammation, enhance immune function, and potentially alter the course of cancer progression.

Immunotherapy, particularly immune checkpoint inhibitors, also shows promise, with gut microbiome composition influencing treatment efficacy. Studies have shown that patients with an increased prevalence of certain beneficial bacterial species, including *Akkermansia muciniphila*, have better responses to PD-1 blockade therapy. This finding indicated that altering the gut microbiome may improve the effectiveness of immunotherapies in treating EC.

Personalized microbiome-based therapies represent the future of oncology, allowing for tailored treatments based on individual microbiota profiles. Advanced sequencing technologies and bioinformatics tools enable the precise characterization of the microbiome, aiding the development of personalized probiotics and dietary treatments designed to modulate the microbiome in a way that supports cancer treatment and prevention. These personalized approaches hold great promise for optimizing therapeutic outcomes and minimizing side effects.

## 9. Recent Bioinformatics Data Related to Microbiome in Endometrial Cancer

Recent bioinformatic analyses have been conducted to deepen the understanding of the microbiome’s role in EC. A study conducted by Li et al. [64] found that *Pelomonas* and *Proteovella* were more abundant and enriched in EC patients with a high tumor burden. By integrating the microbiome data and hematological markers, *Proteovella* was linked to elevated serum D-dimer and fibrin degradation products, both associated with blood clot breakdown. Further analysis identified eight key genes connecting *Proteovella* to fibrin degradation in EC. The combination of *Proteovella*, serum D-dimer, and fibrin degradation products showed high potential for predicting EC onset, with an area under the curve (AUC) of 0.86. These findings suggest that *Prevotella* may contribute to tumor progression by influencing blood clot-related processes.

In another study, they applied a novel analysis pipeline to characterize functionally active microorganisms within EC patients [49]. The study identified over 5000 functionally active microorganisms in the endometrium of EC patients. These active microorganisms were involved in metabolic processes, including the 6-sulfo-sialyl Lewis x epitope and N-acetyl-beta-glucosaminyl, which linked to tumor progression. The host–microbiota interactions were associated with tumor migration and the Apelin signaling pathway. These findings highlight the potential role of active microorganisms in EC development that may be used in future treatment strategies.

Alkhalil et al. [65] analyzed the association between genetic mutations, microbiota, and immune cell infiltration in uterine corpus EC using The Cancer Genome ATLAS (TCGA) database. Thirteen mutant genes commonly found in various cancers were highly prevalent in uterine corpus EC, whereby *PGLYRP2*, *OLFM4*, and *TLR5* were most significant in promoting immune cell invasion. Moreover, several microbiotas, including Firmicutes (*Lachnoclostridium*), Bacteroidetes (*Flammeovirga*), and Proteobacteria (*Ochrobactrum* and *Citrobacter*), have a strong association with the invasion of immune cells, such as memory B cells and regulatory T cells. These results suggest a potential for immunotherapy using targeting bacteria to enhance response in uterine corpus EC.

In another study, they investigated the difference between endometrial microbiota profiles in EC tissues and non-EC tissue [66]. EC tissue exhibited higher bacterial diversity and evenness, with enrichment of *Proteovella*, *Atopobium*, *Anaerococcus*, *Dialister*, *Porphyromonas*, and *Peptoniphilus*. At the same time, *Lactobacillus* and *Gardella* were dominant in both EC and non-EC tissues. This bacterial abundance correlated with clinical factors such as vaginal pH, *Lactobacillus* levels, and EC stage. Another study confirmed that EC tissues exhibit a distinct microbiota distribution with a higher prevalence of pathogenic bacterial genera compared to noncancerous tissues [67]. Despite the overall low microbial abundance in the endometrium, absolute quantification using droplet digital PCR (ddPCR) confirmed the presence of these bacteria. Furthermore, the metabarcoding analysis highlighted site-specific differences in microbiota composition, with *Sphingomonas*, *Acinetobacter*, *Actinomyces*, *Escherichia-Shigella*, *Hydrogenophaga*, *Methylobacterium-Methylorubrum*, *Neisseria*, *Rhodococcus*, and *Rothia* being more specific to the endometrial microbiota in cancerous tissues. These findings suggest a potential link between microbiota dysbiosis and EC.

Other studies have identified microbial signatures associated with endometrial cancer, noting that dysbiosis in the endometrial microbiome may contribute to disease development [43]. They highlighted the lower bacterial load in the cervicovaginal and rectal regions in EC patients, with a depletion of Lactobacillus species (especially *Lactobacillus cripatus*) and an increase in bacterial diversity. They also found that EC was associated with enrichment in specific bacteria, particularly *Porphyromonas*, *Prevotella*, *Peptoniphilus*, and *Anaerococcus*, in the endometrium and lower genital tract. These findings confirmed the importance of microbiome profiling as a tool for early detection and personalized therapeutic strategies in endometrial cancer management.

These bioinformatic approaches provided valuable insights into the complex relationship between the endometrial microbiome and EC, establishing the way for novel diagnostic and therapeutic avenues. Table 2 summarizes the recent bioinformatic studies related to the microbiome in EC.

## 10. Conclusions

A significant gap remains in our understanding of the gut–endometrium axis despite these advancements. The exact mechanism on how the gut microbiome influences the endometrial microbiota and contributes to the pathogenesis of EC is not well understood. Additionally, studies exploring the molecular pathways involved—such as microbial metabolites, immune modulation, and estrogen metabolism—are still lacking, particularly in the context of gut–endometrial microbiota interactions. This gap exists because most of the studies on the gut–endometrial microbiota axis in EC is observational, making it difficult to establish causality. Future research should focus on experimental and longitudinal studies, in order to confirm whether gut and endometrial microbiome alterations contribute to EC progression or are a consequence of the disease. Establishing these precise mechanisms, especially related to microbial metabolites, estrogen metabolism, and immune modulation, could provide critical insights into EC development

Moreover, a longitudinal cohort study that integrates multi-omics approaches, including metabolomic, metagenomic sequencing, and immune profiling, should be conducted to identify specific microbial signatures that are linked to EC risk and progression. By identifying specific microbial signatures of EC, large-scale interventional studies using fecal microbiota transplantation, prebiotics, or probiotics could be conducted to explore the therapeutic potential of microbiome modulation in EC patients. The effect of dietary patterns, hormonal therapies, and antibiotic use on the gut–endometrial axis should also be evaluated to develop personalized prevention strategies. This holistic approach will be essential in formulating targeted therapies that are both effective and personalized.

In conclusion, the interplay between the gut and endometrial microbiome is crucial in the pathogenesis of EC. Targeting these microbial communities offers innovative strategies for prevention and treatment. Continued research into the gut–endometrium axis will provide novel therapeutic strategies, ultimately improving patient outcomes in EC. By enhancing our understanding of the intricate interactions between microbial populations and the host, we can develop more effective, personalized treatments that address the underlying mechanisms of EC and improve overall gynecological health.

## Figures and Tables

**Table 1:** Microbiome-based therapies in endometrial cancer.

Study/Trial Name	Type of Intervention	Patient Population	Key Findings	References
Probiotic Therapy for EC	Probiotics (*Lactobacillus*, *Bifidobacterium*)	EC patients	Restoration of microbial balance, reduction in inflammation, enhanced immune response.	[53,54,55,59]
Fecal Microbiota Transplantation (FMT)	Fecal Microbiota Transplantation	EC patients	Restoration of healthy gut microbiota, reduction in systemic inflammation, modulation of immune responses.	[56]
Immune Checkpoint Inhibitor Therapy	Immune Checkpoint Inhibitors (associated with specific gut bacteria)	EC patients	Patients with higher abundance of *Akkermansia muciniphila* showed better responses to PD-1 blockade therapy.	[57,60]
Antibiotic Therapy	Antibiotic Treatment	EC patients	Selective depletion of harmful bacterial species associated with cancer progression, caution against overuse	[61,62]
Personalized Microbiota- Based Therapy	Personalized Microbiota- Based Therapy	EC patients	Tailored treatments based on individual microbiota profiles, promoting cancer treatment and prevention	[63]

**Table 2:** Summaries of the recent bioinformatic studies related to the microbiome in EC.

Author (Year)	Techniques Used	Sample Size	Key Findings
Li et al. [64] (2021)	16S rRNA sequencing Transcriptome analyses	EC patients = 30 Healthy volunteers = 10	*Pelamonas* and *Prevotella* enriched in EC group *Prevotella* associated with serum D-dimer and fibrin degradation products
Chen et al. [49] (2021)	HMP Unified Metabolic Analysis Network 3 (HUMAnN3) MetaCyc-based GSEA functional enrichment analysis O2PLS model	EC group = 9 Normal subject = 8	More 5000 functionally active microorganisms between EC and normal group These microorganisms involved in many biological processes, including tumor migration and the Apelin signaling pathway
Alkhalil et al. [65] (2024)	Tumor immune estimation resource 2 (TIMER2) CIBERSORT method The Cancer Genomic Atlas (TCGA) database	30 mutant genes	*PGLYRP2*, *OLFM4*, and *TLR5* were most significant in uterine corpus EC Firmicutes (*Lachnoclostridium*), Bacteroidetes (*Flammeovirga*), Proteobacteria (Ochrobactrum, and *Citrobacter*) have strong association with invasion of immune cells, such as memory B cells and regulatory T cells
Wang et al. [66] (2022)	16S rRNA sequencing R language software	28 EC-affected postmenopausal women	*Lactobacillus* and *Gardnerella* were the main bacterial genera present in both EC and adjacent non-EC-invading endometrium *Prevotella*, *Atopobium*, *Anaerococcus*, *Dialister*, *Porphyromonas*, and *Peptoniphilus* were increased in the EC group These endometrial bacteria were associated with vaginal pH, vaginal *Lactobacillus* abundance, and EC clinical stage
Leoni et al. [67] (2024)	Droplet digital PCR Deep metabarcoding NGS	EC patients = 8 Polymyomatous uterus= 6	Site-specific differences in microbiota composition *Sphingomonas*, *Acinetobacter*, *Actinomyces*, *Escherichia–Shigella*, *Hydrogenophaga*, *Methylobacterium–Methylorubrum*, *Neisseria*, *Rhodococcus*, and *Rothia* were specific to the endometrial microbiota in cancerous tissues
Semertzidou et al. [43] (2024)	16S rRNA sequencing	EC group = 37 Benign control group = 24	Lower bacterial load in the cervicovaginal and rectal regions in EC patients, with a depletion in Lactobacillus species EC was associated with an enrichment of specific bacteria, particularly *Porphyromonas*, *Prevotella*, *Peptoniphilus*, and *Anaerococcus*, in the endometrium and lower genital tract.

## Abbreviations

The following abbreviations are used in this manuscript:

EC	Endometrial cancer
FMT	Fecal microbiota transplantation
PCOS	Polycystic ovary syndrome
PI3K	Phosphoinositide 3-kinase
MMR	DNA mismatch repair
HNPCC	Hereditary nonpolyposis colorectal cancer
BPA	Bisphenol A
DES	Diethylstilbestrol
ROS	Reactive oxygen species
SCFAs	Short-chain fatty acids
MAMPs	Microbial-associated molecular patterns
Tregs	Regulatory T cells
NF-κB	Nuclear factor-kappa B
LPS	Lipopolysaccharides
IL-6	Interleukin-6
IL-17	Interleukin-17
TNF-α	Tumor necrosis alpha
AUC	Area under the curve
TCGA	The Cancer Genome Atlas
ddPCR	Digital droplet PCR

## References

[1] Moar K., Pant A., Saini V., Maurya P.K. (2023). Potential biomarkers in endometrial cancer: A narrative review. Biomarkers.

[2] Cronin K.A., Scott S., Firth A.U., Sung H., Henley S.J., Sherman R.L., Siegel R.L., Anderson R.N., Kohler B.A., Benard V.B. (2022). Annual report to the nation on the status of cancer, part 1: National cancer statistics. Cancer.

[3] Siegel R.L., Giaquinto A.N., Jemal A. (2024). Cancer statistics, 2024. CA Cancer J. Clin..

[4] Xu Y., Qiao J. (2022). Association of Insulin Resistance and Elevated Androgen Levels with Polycystic Ovarian Syndrome (PCOS): A Review of Literature. J. Healthc. Eng..

[5] Marin A.G., Filipescu A., Vladareanu R., Petca A. (2024). Metabolic Syndrome and Survival Outcomes in Endometrial Cancer. Cureus.

[6] Shafiee M.N., Ortori C.A., Barrett D.A., Mongan N.P., Abu J., Atiomo W. (2020). Lipidomic Biomarkers in Polycystic Ovary Syndrome and Endometrial Cancer. Int. J. Mol. Sci..

[7] Caretto M., Simoncini T. (2021). Progestin or anti-estrogen treatment for endometrial cancer: Choosing the best option for selected patients. Gynecol. Endocrinol..

[8] Sepich-Poore G.D., Zitvogel L., Straussman R., Hasty J., Wargo J.A., Knight R. (2021). The microbiome and human cancer. Science.

[9] Su X., Gao Y., Yang R. (2022). Gut Microbiota-Derived Tryptophan Metabolites Maintain Gut and Systemic Homeostasis. Cells.

[10] Łaniewski P., Ilhan Z.E., Herbst-Kralovetz M.M. (2020). The microbiome and gynaecological cancer development, prevention and therapy. Nat. Rev. Urol..

[11] Chadchan S.B., Singh V., Kommagani R. (2022). Female reproductive dysfunctions and the gut microbiota. J. Mol. Endocrinol..

[12] Ser H.L., Au Yong S.J., Shafiee M.N., Mokhtar N.M., Ali R.A.R. (2023). Current Updates on the Role of Microbiome in Endometriosis: A Narrative Review. Microorganisms.

[13] Elkafas H., Walls M., Al-Hendy A., Ismail N. (2022). Gut and genital tract microbiomes: Dysbiosis and link to gynecological disorders. Front. Cell Infect. Microbiol..

[14] Baethge C., Goldbeck-Wood S., Mertens S. (2019). SANRA—A scale for the quality assessment of narrative review articles. Res. Integr. Peer Rev..

[15] Mamat Yusof M.N., Chew K.T., Kampan N., Abd Aziz N.H., Md Zin R.R., Tan G.C., Shafiee M.N. (2022). PD-L1 Expression in Endometrial Cancer and Its Association with Clinicopathological Features: A Systematic Review and Meta-Analysis. Cancers.

[16] Tanos P., Dimitriou S., Gullo G., Tanos V. (2022). Biomolecular and genetic prognostic factors that can facilitate fertility-sparing treatment (FST) decision making in early stage endometrial cancer (ES-EC): A systematic review. Int. J. Mol. Sci..

[17] Williams T., Moore J.B., Regehr J. (2023). Polycystic ovary syndrome: Common questions and answers. Am. Fam. Physician.

[18] Shafiee M.N., Seedhouse C., Mongan N., Chapman C., Deen S., Abu J., Atiomo W. (2016). Up-regulation of genes involved in the insulin signalling pathway (IGF1, PTEN and IGFBP1) in the endometrium may link polycystic ovarian syndrome and endometrial cancer. Mol. Cell Endocrinol..

[19] Jamieson A., McAlpine J.N. (2023). Molecular Profiling of Endometrial Cancer from TCGA to Clinical Practice. J. Natl. Compr. Canc Netw..

[20] Mamat @ Yusof M.N., Chew K.T., Kampan N.C., Shafiee M.N. (2023). Expression of PD-1 and PD-L1 in Endometrial Cancer: Molecular and Clinical Significance. Int. J. Mol. Sci..

[21] Yu K., Huang Z.Y., Xu X.L., Li J., Fu X.W., Deng S.L. (2022). Estrogen Receptor Function: Impact on the Human Endometrium. Front. Endocrinol..

[22] Wang Y., Zeng X., Tan J., Xu Y., Yi C. (2022). Diabetes mellitus and endometrial carcinoma: Risk factors and etiological links. Medicine.

[23] Kho P.F., Mortlock S., Yang J., Digna R., Nyegaard M., Low S.-K., Zondervan K.T., Missmer S.A. (2021). Genetic analyses of gynecological disease identify genetic relationships between uterine fibroids and endometrial cancer, and a novel endometrial cancer genetic risk region at the WNT4 1p36. 12 locus. Hum. Genet..

[24] Dörk T., Hillemanns P., Tempfer C., Breu J., Fleisch M.C. (2020). Genetic susceptibility to endometrial cancer: Risk factors and clinical management. Cancers.

[25] Monnin N., Fattet A.J., Koscinski I. (2023). Endometriosis: Update of pathophysiology,(epi) genetic and environmental involvement. Biomedicines.

[26] Yu W., Tu Y., Long Z., Liu J., Kong D., Peng J., Wu H., Zheng G., Zhao J., Chen Y. (2022). Reactive Oxygen Species Bridge the Gap between Chronic Inflammation and Tumor Development. Oxid. Med. Cell Longev..

[27] Costea P.I., Hildebrand F., Arumugam M., Bäckhed F., Blaser M.J., Bushman F.D., de Vos W.M., Ehrlich S.D., Fraser C.M., Hattori M. (2018). Enterotypes in the landscape of gut microbial community composition. Nat. Microbiol..

[28] Sędzikowska A., Szablewski L. (2021). Human Gut Microbiota in Health and Selected Cancers. Int. J. Mol. Sci..

[29] Soni M. (2021). Diet Influences the Gut Microbiota: A Minireview. Bact. Emp..

[30] Ağagündüz D., Özata-Uyar G., Kocaadam-Bozkurt B., Özturan-Şirin A., Capasso R., Al-Assaf S., Özoğul F. (2023). A comprehensive review on food hydrocolloids as gut modulators in the food matrix and nutrition: The hydrocolloid-gut-health axis. Food Hydrocoll..

[31] Merra G., Noce A., Marrone G., Cintoni M., Tarsitano M.G., Capacci A., Lorenzo A.D. (2020). Influence of Mediterranean Diet on Human Gut Microbiota. Nutrients.

[32] Valente A. (2020). The Possible Influence of Microbiota on Food Compulsion. J. Biomed. Sci..

[33] Umu Ö.C.O., Rudi K., Diep D.B. (2017). Modulation of the Gut Microbiota by Prebiotic Fibres and Bacteriocins. Microb. Ecol. Health Dis..

[34] Jasirwan C.O.M., Lesmana C.R.A., Hasan I., Sulaiman A.S., Gani R.A. (2019). The Role of Gut Microbiota in Non-Alcoholic Fatty Liver Disease: Pathways of Mechanisms. Biosci. Microbiota Food Health.

[35] Wilson A.S., Koller K.R., Ramaboli M.C., Nesengani L.T., Ocvirk S., Chen C., Flanagan C.A., Sapp F.R., Merritt Z.T., Bhatti F. (2020). Diet and the Human Gut Microbiome: An International Review. Dig. Dis. Sci..

[36] Hases L., Stepanauskaite L., Birgersson M., Brusselaers N., Schuppe-Koistinen I., Archer A., Engstrand L., Williams C. (2023). High-fat diet and estrogen modulate the gut microbiota in a sex-dependent manner in mice. Commun. Biol..

[37] Li Y., Liu G., Gong R., Xi Y. (2023). Gut Microbiome Dysbiosis in Patients with Endometrial Cancer vs. Healthy Controls Based on 16S rRNA Gene Sequencing. Curr. Microbiol..

[38] Pollet R.M., D’Agostino E.H., Walton W.G., Xu Y., Little M.S., Biernat K.A., Pellock S.J., Patterson L.M., Creekmore B.C., Isenberg H.N. (2017). An Atlas of β-Glucuronidases in the Human Intestinal Microbiome. Structure.

[39] Chen C., Song X., Wei W., Zhong H., Dai J., Lan Z., Li F., Yu X., Feng Q., Wang Z. (2017). The microbiota continuum along the female reproductive tract and its relation to uterine-related diseases. Nat. Commun..

[40] Morańska K., Englert-Golon M., Durda-Masny M., Sajdak S., Grabowska M., Szwed A. (2023). Why Does Your Uterus Become Malignant? The Impact of the Microbiome on Endometrial Carcinogenesis. Life.

[41] Moreno I., Garcia-Grau I., Perez-Villaroya D., Gonzalez-Monfort M., Bahçeci M., Barrionuevo M.J., Taguchi S., Puente E., Dimattina M., Lim M.W. (2022). Endometrial microbiota composition is associated with reproductive outcome in infertile patients. Microbiome.

[42] Fang R.-L., Chen L.-X., Shu W.-S., Yao S.-Z., Wang S.-W., Chen Y.-Q. (2016). Barcoded sequencing reveals diverse intrauterine microbiomes in patients suffering with endometrial polyps. Am. J. Transl. Res..

[43] Semertzidou A., Whelan E., Smith A., Ng S., Roberts L., Brosens J.J., Marchesi J.R., Bennett P.R., MacIntyre D.A., Kyrgiou M. (2024). Microbial signatures and continuum in endometrial cancer and benign patients. Microbiome.

[44] Lu W., He F., Lin Z., Liu S., Tang L., Huang Y., Hu Z. (2021). Dysbiosis of the endometrial microbiota and its association with inflammatory cytokines in endometrial cancer. Int. J. Cancer.

[45] Stabile G., Doria A., Bruno M., D’Indinosante M., Gallotta V., Fanfani F., Scambia G., Restaino S., Vizzielli G., Carlucci S. (2024). The Role of the Endometrial Microbiota in Endometrial Cancer: A Systematic Review of the Literature. J. Clin. Med..

[46] Sola-Leyva A., Andrés-León E., Molina N.M., Terron-Camero L.C., Plaza-Díaz J., Sáez-Lara M.J., Gonzalvo M.C., Sánchez R., Ruíz S., Martínez L. (2021). Mapping the entire functionally active endometrial microbiota. Human. Reprod..

[47] Walsh D.M., Hokenstad A.N., Chen J., Sung J., Jenkins G.D., Chia N., Nelson H., Mariani A., Walther-Antonio M.R. (2019). Postmenopause as a key factor in the composition of the Endometrial Cancer Microbiome (ECbiome). Sci. Rep..

[48] Gilbert J.A., Blaser M.J., Caporaso J.G., Jansson J.K., Lynch S.V., Knight R. (2018). Current understanding of the human microbiome. Nat. Med..

[49] Chen P., Guo Y., Jia L., Wan J., He T., Fang C., Li T. (2021). Interaction Between Functionally Activate Endometrial Microbiota and Host Gene Regulation in Endometrial Cancer. Front. Cell Dev. Biol..

[50] Wang R., Yang X., Liu J., Zhong F., Zhang C., Chen Y., Sun T., Ji C., Ma D. (2022). Gut microbiota regulates acute myeloid leukaemia via alteration of intestinal barrier function mediated by butyrate. Nat. Commun..

[51] Sinha S.R., Haileselassie Y., Nguyen L.P., Tropini C., Wang M., Becker L.S., Sim D., Jarr K., Spear E.T., Singh G. (2020). Dysbiosis-Induced Secondary Bile Acid Deficiency Promotes Intestinal Inflammation. Cell Host Microbe.

[52] Feng Q., Chen W.-D., Wang Y.-D. (2018). Gut microbiota: An integral moderator in health and disease. Front. Microbiol..

[53] Ma W., Mao Q., Xia W., Dong G., Yu C., Jiang F. (2019). Gut microbiota shapes the efficiency of cancer therapy. Front. Microbiol..

[54] Alli S.R., Gorbovskaya I., Liu J.C.W., Kolla N.J., Brown L., Müller D.J. (2022). The Gut Microbiome in Depression and Potential Benefit of Prebiotics, Probiotics and Synbiotics: A Systematic Review of Clinical Trials and Observational Studies. Int. J. Mol. Sci..

[55] Gibson G.R., Hutkins R., Sanders M.E., Prescott S.L., Reimer R.A., Salminen S.J., Scott K., Stanton C., Swanson K.S., Cani P.D. (2017). Expert consensus document: The International Scientific Association for Probiotics and Prebiotics (ISAPP) consensus statement on the definition and scope of prebiotics. Nat. Rev. Gastroenterol. Hepatol..

[56] De Groot P.F., Frissen M., De Clercq N., Nieuwdorp M. (2017). Fecal microbiota transplantation in metabolic syndrome: History, present and future. Gut Microbes.

[57] Gopalakrishnan V., Spencer C.N., Nezi L., Reuben A., Andrews M.C., Karpinets T.V., Prieto P., Vicente D., Hoffman K., Wei S.C. (2018). Gut microbiome modulates response to anti–PD-1 immunotherapy in melanoma patients. Science.

[58] Sui Y., Wu J., Chen J. (2021). The role of gut microbial β-glucuronidase in estrogen reactivation and breast cancer. Front. Cell Dev. Biol..

[59] Plaza-Diaz J., Ruiz-Ojeda F.J., Gil-Campos M., Gil A. (2019). Mechanisms of action of probiotics. Adv. Nutr..

[60] Routy B., Le Chatelier E., Derosa L., Duong C.P., Alou M.T., Daillère R., Fluckiger A., Messaoudene M., Rauber C., Roberti M.P. (2018). Gut microbiome influences efficacy of PD-1–based immunotherapy against epithelial tumors. Science.

[61] Alexander J.L., Wilson I.D., Teare J., Marchesi J.R., Nicholson J.K., Kinross J.M. (2017). Gut microbiota modulation of chemotherapy efficacy and toxicity. Nat. Rev. Gastroenterol. Hepatol..

[62] Elkrief A., Derosa L., Zitvogel L., Kroemer G., Routy B. (2019). The intimate relationship between gut microbiota and cancer immunotherapy. Gut Microbes.

[63] Bullman S., Pedamallu C.S., Sicinska E., Clancy T.E., Zhang X., Cai D., Neuberg D., Huang K., Guevara F., Nelson T. (2017). Analysis of Fusobacterium persistence and antibiotic response in colorectal cancer. Science.

[64] Li C., Gu Y., He Q., Huang J., Song Y., Wan X., Li Y. (2021). Integrated Analysis of Microbiome and Transcriptome Data Reveals the Interplay Between Commensal Bacteria and Fibrin Degradation in Endometrial Cancer. Front. Cell. Infect. Microbiol..

[65] Alkhalil S.S., Almanaa T.N., Altamimi R.A., Abdalla M., El-Arabey A.A. (2024). Interactions between microbiota and uterine corpus endometrial cancer: A bioinformatic investigation of potential immunotherapy. PLoS ONE.

[66] Wang L., Yang J., Su H., Shi L., Chen B., Zhang S. (2022). Endometrial microbiota from endometrial cancer and paired pericancer tissues in postmenopausal women: Differences and clinical relevance. Menopause.

[67] Leoni C., Vinci L., Marzano M., D’Erchia A.M., Dellino M., Cox S.N., Vitagliano A., Visci G., Notario E., Filomena E. (2024). Endometrial Cancer: A Pilot Study of the Tissue Microbiota. Microorganisms.

